# Peripheral Arteriovenous Malformation Embolization Using Squid

**DOI:** 10.1155/2023/8858656

**Published:** 2023-09-30

**Authors:** Saima Ahmad, Moeez Uddin

**Affiliations:** ^1^Diagnostic and Interventional Neuroradiology Department, Lahore General Hospital, Lahore, Pakistan; ^2^Lancashire Teaching Hospitals NHS Foundation Trust, UK

## Abstract

**Materials and Methods:**

Between January 2018 and December 2020, twenty patients (7 men and 13 women) with peripheral high-flow arteriovenous malformations who were treated primarily with arterial embolization using squid were retrospectively included. Anatomical sites being treated included the head and neck (16), extremities (2), uterus (1), and pelvis (1). Squid was used as the sole embolic agent in 15 patients, and transarterial embolization was employed in all cases except one where direct puncture embolization was used. Treatments were delivered over one or two sessions, with or without surgery. A total of 27 sessions were carried out with an interval time ranging from 6 to 36 months between sessions.

**Results:**

Technical success was achieved in all cases. In those patients treated with squid alone, 13 exhibited total devascularization following embolization, and a further 4 required surgical excision to achieve complete obliteration of the arteriovenous malformation. There were no major complications, cases of microcatheter entrapment, or dimethyl sulfoxide-related pain recorded. On follow-up, one patient reported persistent pain, and another patient developed a garlicky taste. All other patients reported complete resolution of symptoms following treatment.

**Conclusion:**

This study demonstrates the successful use of squid in managing peripheral arteriovenous malformations with low complication rates and long-term stable results, therefore validating its efficacy when used alone or in combination with other embolic agents. Squid may be the preferred embolic agent in any interventional radiologist's armamentarium as it offers formulations with varying viscosities (squid-18 and squid-12). We conclude that squid should be considered as a first-line embolic agent in the management of peripheral arteriovenous malformations.

## 1. Introduction

Arteriovenous malformations (AVMs) are vascular lesions that can develop in any part of the body and are characterized by the presence of arteriovenous microfistulae through a vascular nidus. They have a congenital etiology, and the location, scale, and degree of arteriovenous shunting through the lesion determine the symptomatology and clinical features of AVMs [1]. Peripheral AVMs can be extremely challenging to manage, and contemporary treatment strategies increasingly utilize endovascular embolization as the modality of choice [2]. In such cases, where embolization is the primary treatment method, obliteration rates of less than 50% have been reported [3].

The use of ethylene-vinyl alcohol (EVOH) copolymers was first reported by Akmangit et al. and paved the way for innovative embolic agents to be developed in the management of peripheral AVMs [3, 4]. Onyx is an EVOH-based embolic agent that, due to its slow polymerization and nonadhesive properties, was frequently used as it facilitated longer embolization intervals during the procedure [3].

Similarly, squid is another nonadhesive EVOH-based liquid embolic agent that is available in two versions (squid-12 and squid-18). Squid-12 has been shown to have an added advantage when utilized for embolization of AVMs due to its lower viscosity and higher distal penetration [5]. The essential difference with squid is the smaller size of the grains of tantalum powder. This smaller grain size is aimed at enhancing the homogeneity in radiopacity and improving the visibility during longer injection times [5].

In this study, we present our treatment experience with using squid as the primary embolic agent in 20 patients who presented with peripheral AVMs.

## 2. Materials and Methods

A retrospective analysis of patients who were treated for peripheral AVMs with embolization using squid alone or in conjunction with other embolic agents between January 2018 and December 2020 was conducted. 20 patients (male and female) with ages ranging from 5 to 45 years were identified and included in the study.

All cases were discussed and managed by a multidisciplinary team comprising of plastic surgeons, interventional radiologists, and dermatologists. Prior to referral, patients had already undergone computerized tomography angiography (CTA), magnetic resonance imaging (MRI), or magnetic resonance angiography investigations. The patient's presenting complaint, angioarchitecture on digital subtraction angiography (DSA), and contrast-enhanced tests were used to reach a multidisciplinary consensus on whether embolization could be followed by surgery or not. All patients were counseled comprehensively about the proposed treatment and its possible side effects to ensure informed consent. The choice of embolizing material depends upon the availability and the type of AVM.

Data regarding the anatomical site, clinical symptoms, history of prior treatments, number of embolization sessions needed, embolic agents used, technical outcomes of procedures, and complications was extracted for analysis in the study. Patients were followed up for an average period of 6-36 months, and outcomes were assessed using clinical examination, review of symptoms (relief, persistence, or aggression), follow-up imaging (MRI/CTA), and delayed complications.

### 2.1. Embolization Technique

All squid embolization sessions were performed under general anaesthesia on a biplane angiography unit. In our unit, embolization is normally delivered as an adjuvant treatment with a view to reduce the size of the AVM nidus and obliterate fistulous components, facilitating definitive surgical excision and total cure in a few selected cases.

Vascular access was obtained through the transfemoral route for all procedures. Treatment was preceded by diagnostic angiography to analyse the feeders in difficult cases; however, in most cases, the diagnostic and therapeutic procedure was done in the same session. A guiding catheter was used as an intervention in every procedure. The microcatheter–wire assembly was navigated under roadmap guidance, commonly into the most accessible dominant feeder supplying the nidus. Once the distal position was achieved, the flow was assessed with superselective angiograms, and the microcatheter position was further refined to achieve the wedge position. All microcatheters that were used were dimethyl sulfoxide (DSMO) compatible both with detachable and nondetachable tips. All microcatheters were navigated using microwires of size 0.08. If wedging did not occur, we created a pseudowedge effect by creating a small plug of squid around the microcatheter tip to prevent reflux. Embolization was continued as long as nidal percolation occurred without the embolic material entering into the draining vein or refluxing around the microcatheter tip. When either of these occurred, the flow was redirected by briefly pausing the injection for an interval of fewer than 2 minutes and then resumed. Multiple feeders were accessed in the same or consequent procedures. Embolization was terminated if complete embolization was achieved angiographically, and significant reflux occurred around the microcatheter tip or further embolization of the AVM was deemed risky. The microcatheter was retrieved using controlled traction.

### 2.2. Squid Administration Modality

Squid, a liquid embolic agent based on ethylene-vinyl alcohol, was used for embolization alone or in addition to other embolic agents. Variations of squid which were administered included squid-18 and squid-12 which exhibit high and low viscosity, respectively. To increase the embolizing force, squid was either used in conjunction with other embolizing agents (PVA (polyvinyl alcohol) particles/Bleomycin) or alone. The squid was constantly combined with a “shaker unit” (time = 20 minutes) up to the point of use in order to create a fluoroscopically visible tantalum suspension. DMSO-compatible microcatheters were used for administration, with the dead space in the microcatheter being filled with DMSO before squid infusion. The squid was injected into the target vessel with a 1 ml syringe for 45 seconds to replace the DMSO in the microcatheter's dead space, followed by gradual retrieval to avoid trapping. It was injected slowly (60–90 s) to mitigate DMSO toxicity and preserve tolerability even though thromboembolism of small vessels.

## 3. Results

A total of 20 patients were taken up for embolization of peripheral AVMs between Jan 2018 and Dec 2020. Of these, 16 patients had head and neck AVMs, 2 AVMs on extremities, 1 uterine, and 1 at the pelvic location. 13 patients were female, and 7 patients were male with ages ranging from 5 to 45 years. The most common presentation was that of pulsatile swelling followed by pain, cosmetic disfigurement, and haemorrhage in descending order.

Squid-12 and 18 were the sole liquid embolic agents used in 15 cases. In the other 5 cases, additional embolic agents such as PVA particles and coils were also used. Both detachable and conventional DMSO-compatible microcatheters were used in all procedures. In one case, percutaneous puncture embolization was also required. A total of 27 sessions of embolizations were performed. The average volume of squid used in each case was 2.5 ml, and the average duration of injection was 25 minutes.

13 AVMs were completely obliterated by sole embolization with squid, and a further 4 AVMs were almost completely obliterated with surgical excision following embolization. In 2 cases, there was partial embolization, but they refused further treatment, and in a single case, there was temporary relief followed by recurrence (see Table 1 for a summary of results)

### 3.1. Illustrative Cases

#### 3.1.1. Case 1

A 21-year-old female with a facial AVM presented with swelling affecting the left cheek. The swelling was associated with headache and episodic visual blurring in the left eye only. MRI was performed and showed features suggestive of a facial AVM. After a detailed discussion with the patient, elective embolization was done. The AVM was fed by branches of the external carotid artery—internal maxillary artery, which was selected by a DMSO-compatible microcatheter. The microcatheter was positioned distally in the feeding vessel, proximal to the AVM nidus, and embolized with squid. The squid cast was allowed to harden for 20 minutes. Multiple intraembolization angiograms were performed to see the extension of squid cast into the feeding vessel and early draining vein. Each arteriogram demonstrated less shunting until the whole AVM was embolized. The microcatheter was retrieved uneventfully after the procedure, and the control angiogram showed total obliteration of the AVM.

**Figure 1:**
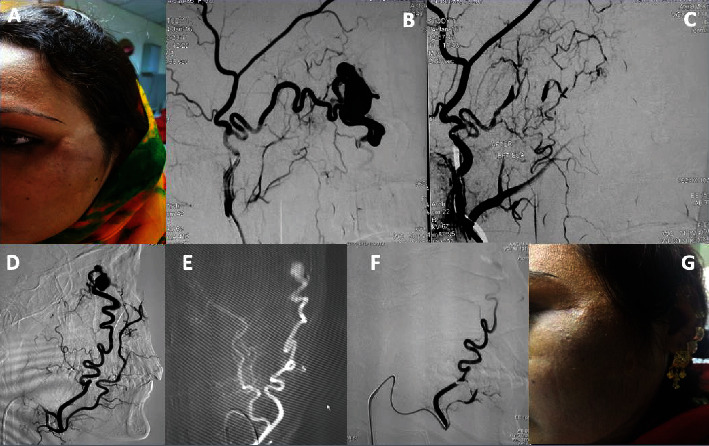
A 21-year-old female with left facial AVM was presented with swelling left cheek. The swelling was associated with headache and episodic visual blurring in the left eye only. (A) Preprocedure photograph. (B) Selective left ECA angiogram revealed dilated anomalous vascular channels fed by the distal internal maxillary artery. (C) Postembolization angiogram using squid-18, showing total obliteration of nidus. (D) DSA after 9 months showing reconstitution of the previous channel through linguofacial trunk. (E) Roadmap. (F) Postembolization angiogram after the second session showing complete obliteration. (G) Follow-up after 2 years.

Nine months following the initial procedure, a follow-up angiogram showed reconstitution of the left distal internal maxillary artery by the linguofacial trunk and a residual fistulous type of AVM. The second session embolization was done with squid-18 which lead to the complete resolution of symptoms, and the patient remained symptom-free over a 3-year follow-up period (Figure 1).

#### 3.1.2. Case 2

A 30-year-old female presented with a tender lump on the dorsum of her right foot that had been present for years. It had become extremely painful in recent months which resulted in the patient walking with a limp. Physical examination revealed a slightly elevated dorsum of the foot, and subsequent magnetic resonant imaging showed a focal vascular lesion with flow voids identified on T1 and T2 sequences. A diagnostic angiogram was performed, and treatment was planned in the same session. It was a high-flow AVM with feeders arising from several branches of the anterior and posterior tibial arteries (ATA and PTA), as well as rapid shunting within the lesion and outflow into enlarged anterior tibial veins. A microcatheter microwire assembly, which was DMSO compatible, was navigated from the left common femoral artery to the right popliteal artery. Both ATA and PTA were engaged super selectively, and squid-12 was administered in a pulsatile fashion until no more shunting was evident, and a small amount of squid was refluxed into the proximal draining veins.

**Figure 2:**
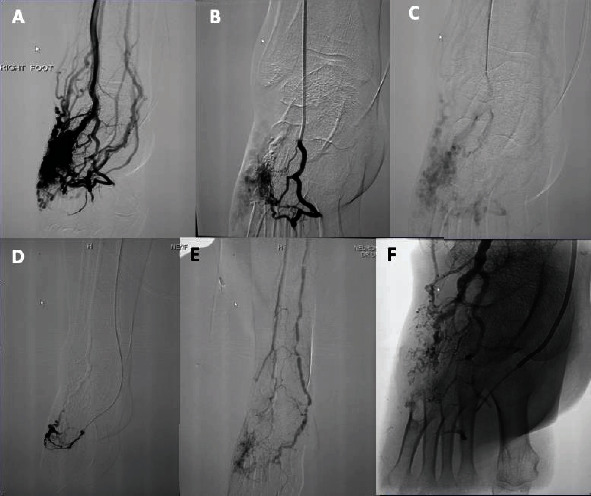
A 30-year-old female presented with a painful lump on the dorsum of her right foot which had been present for several years. In the past several months, it had become increasingly painful, and now, she walked with a slight limp. (A) Selective right foot angiogram showing shunting and rapid egress of contrast from the arteriovenous malformation through the anterior tibial artery and posterior tibial artery. (B, C) Superselective angiogram with microcatheter. (D) Superselective angiogram with microcatheter showing residual flow from posterior tibial artery. (E) Postembolization digital subtraction angiogram shows complete occlusion of the AVM with normal perfusion of the foot. (F) Therapeutic embolization, static fluoroscopic image shows squid filling the lesion.

Postembolization angiography revealed total obliteration of the AVM and excellent perfusion of the remainder of the foot. The patient made an uneventful recovery and was symptom-free at 1-year follow-up (Figure 2).

#### 3.1.3. Case 3

A 45-year-old female with an insignificant medical history presented with a progressively increasing swelling involving the upper lip, left cheek, and ipsilateral mandible. Bruit was evident on palpation of the lesion, and CTA revealed a large facial AVM with multiple feeders arising from the left external carotid artery. Following a multidisciplinary review, an endovascular procedure was planned because of the high risk of intraoperative haemorrhage.

**Figure 3:**
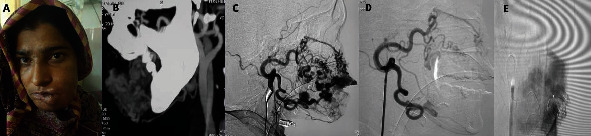
A 45-year-old female with insignificant medical history presented with progressively increasing swelling involving, upper lip, left cheek, and left side of the mandible. (A) Preembolization photograph showing extensive growth involving left lower half of face and lips. (B) Computed tomographic angiographic reformat of the left mandible area showing enlarged vascular channels. (C) Selective left external carotid artery angiogram, lateral projection, showing AVM feeding by linguofacial trunk and internal maxillary artery. (D) Postembolization, left external carotid artery angiogram showing significant reduction in the flow of nidus. (E) Superselective angiogram with microcatheter during embolization showing squid cast.

A head and neck DSA was performed which revealed multiple feeders, including the internal maxillary artery and linguofacial trunk on the left side, draining into superficial veins. Feeding arteries were super selectively catheterized, and both squid-18 and 12 were used for embolization. Postembolization angiogram confirmed complete obliteration of the AVM, and the patient's clinical symptoms resolved completely after a month. The patient remained symptom-free at the 12-month follow-up however refused cosmetic surgery to manage the residual disfigurement (Figure 3).

## 4. Discussion

AVMs are congenital in nature, and they can occur in any anatomical location and can be surrounded by bony or soft tissue coverage [6]. AVMs present with a variety of clinical signs and symptoms, ranging from a minor asymptomatic lesion to a serious injury that impairs vital functions. They usually manifest as pulsatile masses with elevated temperature which can also become infected with fremitus. The Cho scale, which is based on the angioarchitecture created by the nourishing arteries, nidus, and drainage veins, is used to classify peripheral AVMs and forms the basis for therapeutic planning and prognostication [7]. AVMs with multiple arterial branches that run through a single vein, types 1 and 2, exhibit a greater response to treatment, according to Cho et al. [7]. Type 3a and 3b AVMs, which have several inflows and outflows, have the worst response [8].

Yakes developed a new classification system for such lesions' angioarchitecture. Endovascular methods and embolic agents that can successfully ablate these AVMs are determined using the AVM Classification System. Yakes' classification contained lesions that, according to the International Society for the Study of Vascular Anomalies classification 2018, are considered arteriovenous fistulas (AVFs) rather than arteriovenous malformations (AVMs).

A validated therapeutic alternative is endovascular transcatheter embolization of peripheral AVMs prior to surgical excision in selected cases. A variety of embolic agents can be utilized in this technique, and multiple sessions are often required to achieve the desired outcome [1].

Liquid embolic agents seem to be the most appropriate for AVMs because of their capacity to form a cast that penetrates the nidus and occludes the various feeders [1]. The safety and efficacy of EVOH for the treatment of extracranial AVMs have been widely validated in the literature. Onyx-18, and EVOH copolymer liquid embolic agent, is said to efficiently infiltrate the nidal compartment and create an embolic cast adjacent to the nidus [5].

In this report, we presented our first clinical experience with squid, a new EVOH copolymer. Squid has recently been launched in four separate formulations, i.e., 18, 18LD, 12, and 12LD. The LD (low density) models contain 30% lesser tantalum, which can aid in X-ray visualization of structures hidden behind thick embolic casts. The micronized tantalum particles in squid are smaller than those of Onyx, resulting in a more homogeneous solution. In this study, we only used the 12 and 18 formulations to resolve the target lesions. In general, squid-18 was chosen for initial plug forming in the embolized feeder, and injection was started with squid-12 because it penetrates the embolic cast more effectively than squid-18. Squid-18 behaved similarly to Onyx 18 in our view.

Squid's lower tantalum concentration (30%), which may improve vascular visibility during embolization and minimize metallic artifacts during imaging follow-up, is another advantage over Onyx [9, 10].

The squid was often infused into DMSO-compatible microcatheters, with the dead space in the microcatheter being filled with DMSO before the infusion. Using a 1 ml syringe, the squid was pumped for 45 seconds to replace the DMSO in the dead space of the microcatheter and then into the target vessel, gradually retrieving the microcatheter to prevent trapping. To reduce DMSO toxicity and ensure tolerability, the squid was injected slowly over 60–90 seconds [11].

Following intra-arterial injection, DMSO has been shown to cause an inflammatory response, vasospasm, and endothelial necrosis. It is worth noting that these findings are seen in studies using higher amounts and infusion speeds than what is usually used for cerebral Onyx embolization. Although DMSO metabolites are mostly excreted through the kidneys, they are also excreted through the lungs which may lead to possible pulmonary toxicity [12].

A few other complications related to squid embolization are a slow setting time (1-3 min), catheter retention, pigmentation, and high material cost. The injection is also painful, so general anaesthesia is mandatory [11]. However, in our case series, no side effects of DMSO toxicity were observed, and there was no catheter entrapment postembolization. The only significant side effect which was observed in most cases was postoperative pain.

Another advantage of squid is the presence of fewer beam artifacts, which helps to rule out the exact size of residual AVM in those cases where we intend to do radiosurgery after initial embolization. When compared to Onyx, the initial clinical encounter of squid reveals subjectively decreased artifacts on postembolization CT scans. On CT and flat-panel CT acquisitions, all four squid versions caused less beam hardening artifacts compared to Onyx 18. Squid LD variants generated fewer objects than their standard density counterparts [10].

## 5. Conclusion

Transcatheter embolization with squid, with or without postembolization surgical excision, is a promising treatment choice for peripheral AVMs. Thanks to its slow polymerization, squid seems to have controlled embolization which allows for deep penetration in the nidus with less chance of catheter gluing due to its nonadhesive properties. There is a paucity of evidence in the current literature regarding the use of squid in the management of peripheral AVMs, and therefore, more research will be required to further characterize its safety profile in the peripheral vasculature.

## Figures and Tables

**Table 1:** Patients' age, sex, AVM location, clinical presentation, previous interventions, arterial feeders, no. of embolization sessions, concentration of squid used, other embolic agent used, complications, technical and clinical results, and follow-up duration.

Patient no.	Age/sex	Lesion site	Previous interventions	Arterial feeders	No. of embolization sessions	Conc. of squid used	Other embolic agents used	Technical results	Clinical results	Follow-up months	Outcome
1	19/F	L auricular	Surgery two years ago	L posterior auricular artery	1	18, 12	No	Complete	Complete	12	Good
2	14/F	R auricular, scalp & neck	No	L posterior auricular artery, Occipital artery	1	18	PVA particles (350-500u)	Partial	Temporary relief/recurrence	16	Persistence of pain & residual
3	12/M	R mandibular	Surgery six months ago	R lingual-facial trunk	2	18	PVA particles (250-350u)	Complete	Success	8	Operated, good
4	11/M	R mandibular	Surgery one year ago	R lingual-facial trunk	2	18	No	Complete	Success	10	Operated, good
5	23/M	Nape and left side neck	No	Bilateral occipital arteries & bilateral superficial temporal arteries	2	18	No	Partial 80%/ICA feeders not embolized	Partial	12	Operated, Good
6	35/F	R auricular	No	Right posterior auricular artery	2	18, 12	No	Complete	Success	18	Satisfactory/cosmetic issue
7	28/F	Nasal root and bridge	No	L internal maxillary artery, L ophthalmic artery	1	18	No	Complete	Success	16	Operated twice, good
8	16/F	L auricular & scalp	Two surgeries with 5-year interval	B/L occipital arteries & superficial temporal arteries, L posterior auricular artery, R vertebral artery	1	18, 12	PVA particles (250-350u), NBCA	Partial 80% improved	Partial	24	Good
9	30/F	R auricular	No	R posterior auricular artery	1	18	No	Complete	Success	22	Good
10	42/M	R auricular, preauricular	No	R posterior auricular artery & occipital artery	1	18	No	Complete	Success	20	Good
11	5/M	R facial	No	R internal maxillary artery	1	18	Coils	Complete	Success	36	Good
12	21/F	L temple	No	L internal maxillary artery	2	18	No	Complete	Success	30	Satisfactory/cosmetic issue
13	25/F	L mandible, lip	No	L internal maxillary artery & lingual-facial trunk	1	18, 12	PVA particles (350-500u)	Partial 60% embolized	Partial	24	Satisfactory/cosmetic issue
14	11/F	R facial	Surgery 5 years ago	R internal maxillary artery	2	18	PVA particles (250-350u)	Partial 80% embolized	Partial	24	Satisfactory/cosmetic issue
15	45/F	L Ala of nose, upper lip, face	No	L internal maxillary artery & lingual-facial trunk, L ophthalmic artery	2	18	No	Partial	Partial	26	Good
16	20/M	L temple	No	L internal maxillary artery	1	18	No	Complete	Success	12	Good
17	30/F	Uterine	No	L uterine artery	1	18	No	Complete	Success	18	Good
18	29/M	Natal cleft & scrotal region	No	L internal iliac artery-multiple branches	1	18	No	Partial 50% embolized	Partial	24	Good
19	30/F	R foot	No	R anterior tibial artery & posterior tibial artery	1	18	PVA particles (250-350u), NBCA	Complete	Success	26	Good
20	37/F	L arm	No	L radial artery	1	18	No	Complete	Success	28	Good
